# ZebraReg—a novel platform for discovering regulators of cardiac regeneration using zebrafish

**DOI:** 10.3389/fcell.2024.1384423

**Published:** 2024-05-10

**Authors:** Kateřina Apolínová, Ferran Arqué Pérez, Sylvia Dyballa, Benedetta Coppe, Nadia Mercader Huber, Javier Terriente, Vincenzo Di Donato

**Affiliations:** ^1^ ZeClinics SL, Barcelona, Spain; ^2^ Biomedicine, Department of Medicine and Life Sciences, Faculty of Health and Life Sciences, Pompeu Fabra University, Barcelona, Spain; ^3^ ZeCardio Therapeutics SL, Barcelona, Spain; ^4^ Developmental Biology and Regeneration, Institute of Anatomy, University of Bern, Bern, Switzerland; ^5^ Department for Biomedical Research DBMR, University of Bern, Bern, Switzerland; ^6^ Centro Nacional de Investigaciones Cardiovasculares CNIC, Madrid, Spain

**Keywords:** heart regeneration, zebrafish, genetic ablation, cardiomyocyte, proliferation, drug discovery, target validation

## Abstract

Cardiovascular disease is the leading cause of death worldwide with myocardial infarction being the most prevalent. Currently, no cure is available to either prevent or revert the massive death of cardiomyocytes that occurs after a myocardial infarction. Adult mammalian hearts display a limited regeneration capacity, but it is insufficient to allow complete myocardial recovery. In contrast, the injured zebrafish heart muscle regenerates efficiently through robust proliferation of pre-existing myocardial cells. Thus, zebrafish allows its exploitation for studying the genetic programs behind cardiac regeneration, which may be present, albeit dormant, in the adult human heart. To this end, we have established ZebraReg, a novel and versatile automated platform for studying heart regeneration kinetics after the specific ablation of cardiomyocytes in zebrafish larvae. In combination with automated heart imaging, the platform can be integrated with genetic or pharmacological approaches and used for medium-throughput screening of presumed modulators of heart regeneration. We demonstrate the versatility of the platform by identifying both anti- and pro-regenerative effects of genes and drugs. In conclusion, we present a tool which may be utilised to streamline the process of target validation of novel gene regulators of regeneration, and the discovery of new drug therapies to regenerate the heart after myocardial infarction.

## 1 Introduction

Cardiovascular diseases, especially myocardial infarction (MI), remain the leading cause of death worldwide, and their prevalence is predicted to increase by 130% by 2030 (World Health Organization, 2014). An MI is a critical cardiovascular event characterised by the sudden interruption of blood supply to a portion of the heart muscle, leading to the irreversible death of cardiomyocytes close to the injury zone and their replacement with a non-contractile scar (Wang et al., 2023). This detrimental process, known as cardiac remodelling, can result in severe and potentially life-threatening complications such as impaired cardiac function, heart failure, arrhythmias, or cardiac arrest (Cohn et al., 2000; Yamaguchi et al., 2008). The limited regenerative capacity of the adult mammalian heart leads to the irreversible loss of functional myocardium following injury (Wang et al., 2023). While the human heart possesses some regenerative potential (Bergmann et al., 2009), this ability is insufficient to replace the vast number of lost cardiomyocytes and restore cardiac function fully. Consequently, exploring strategies to enhance heart regeneration through the activation of the proliferative program in pre-existing cardiomyocytes has become a topic of great interest in the field of regenerative and cardiovascular medicine (Laflamme and Murry, 2011; Garbern and Lee, 2022).

Understanding the fundamental cellular and molecular mechanisms underlying heart regeneration is crucial for developing effective therapeutic interventions (Sadek and Olson, 2020). Several organisms, including zebrafish, newts, and neonatal mice, exhibit remarkable regenerative abilities, also in the context of cardiac injury (Gamba et al., 2014). Zebrafish (*Danio rerio*) has emerged as an advantageous vertebrate model organism due to its extraordinary regenerative capacity (Marques et al., 2019). Upon cardiac injury, zebrafish can efficiently regenerate damaged myocardium, restore cardiac function, and exhibit complete structural and functional recovery (Poss et al., 2002), providing a unique opportunity to identify and validate potential modulators of heart regeneration. Several other characteristics, such as small size, availability of genetic manipulation methods, relative simplicity of the cardiovascular system, and a high degree of genetic conservation between zebrafish and humans have cemented the zebrafish as a beneficial model for cardiac research (González-Rosa, 2022).

In particular, zebrafish larvae – developmental stage spanning from 72 h post fertilization (hpf) to 29 days post fertilization (dpf) - possess numerous advantages in the context of cardiac research. Analogously to humans (Sylva et al., 2014), the heart is the first organ to develop in the zebrafish embryo; it starts exhibiting contractile activity as soon as 24 hpf (Glickman and Yelon, 2002) and adopts an adult-like morphology and electrical function by 72 hpf (Matrone et al., 2013). From an electrophysiological point of view, the heart is mature with a stable heartbeat from 96 hpf onwards (Tessadori et al., 2012). Thanks to their transparency and availability of transgenic methods allowing for heart-specific expression of fluorophores, visualisation of the heart during the early stages of development can be performed with ease (González-Rosa, 2022). Perhaps, most importantly, zebrafish larvae, with their small size and rapid development, are exceptionally well-suited for large-scale, high-throughput screens that can expedite the discovery of novel therapeutic agents (Lessman, 2011; Kithcart and MacRae, 2018).

In order to study heart regeneration in the zebrafish, a myocardial injury must first be induced. Ideally, the injury should be easy to perform, reproducible, and mimic the pathophysiology of a human MI. The first injury model reported consisted of a ventricular apex resection with fine scissors, leading to an amputation of 20%–25% of the ventricle (Poss et al., 2002). However, this injury model presents several disadvantages, including a relative variability in injury size, being technically challenging, and not inducing any scarring, leading to it being not closely similar to the pathophysiology of a human MI (Ross Stewart et al., 2022). The limitations of this system led to the emergence of cryoinjury as an alternative model (Chablais et al., 2011; González-Rosa et al., 2011; Schnabel et al., 2011). In this injury model, the surface of the ventricle is frozen by applying a cryoprobe. As a result, ∼25% of the ventricle is damaged through the cryoinjury, which affects all cell types present at the site of the injury. Unlike ventricular resection, cryoinjury induces fibrosis, necrotic cell death, and enucleation of cardiomyocytes, making it pathophysiologically most similar to human MI (Ross Stewart et al., 2022). However, both methods are technically challenging and require an open chest surgery. Additionally, they are limited to adult animals and cannot be performed on a high-throughput scale (González-Rosa, 2022).

Cardiac injury performed at the larval stage is however more convenient to increase throughput. Currently, two injury models are available in the zebrafish larva: laser ablation and genetic ablation (Ross Stewart et al., 2022). Laser ablation induces a highly localised injury to the ventricle and can be performed at a relatively high-throughput scale of up to 50–60 larvae per hour (Matrone et al., 2013); however, a specialised laser is required (Ross Stewart et al., 2022). Genetic ablation is based on a transgenic system inducing cell type-specific expression of nitroreductase (NTR), an *E. coli* enzyme, which catalyses the conversion of the nontoxic pro-drug Metronidazole (MTZ) into a cytotoxic DNA cross-linking agent (Curado et al., 2007). Cell death is limited to NTR-expressing cells thanks to the intracellular restricted localization of the cytotoxic agent. This system thus provides a temporally and spatially controlled cell type-specific ablation. A major advantage of the system is its adaptability to use in high-throughput screens, as virtually hundreds or thousands of embryos can be easily treated simultaneously by dissolving MTZ in the zebrafish embryo medium (González-Rosa, 2022). An injury of >60% of the ventricular myocardium is tolerated using this method and is efficiently repaired through myocardial regeneration (Wang et al., 2011). Regeneration after genetic ablation is faster than in other injury models. In fact, it has been observed that because of the targeted death of cardiomyocytes, a faster recovery is induced by the uninjured endocardium and epicardium (Wang et al., 2011).

In this manuscript, we introduce ZebraReg, a novel, versatile method that combines genetic and pharmacological techniques to achieve the specific ablation of a specific sub-population of ventricular cardiomyocytes in zebrafish larvae and a medium-throughput imaging-based method for the longitudinal analysis of the regenerative process. The system relies on a double transgenic system combining the NTR-MTZ system with an inducible Cre recombinase to facilitate the specific ablation of *tbx5a*-positive ventricular cardiomyocytes corresponding to the first heart field (FHF) (Sánchez-Iranzo et al., 2018). The FHF is targeted as it represents the larger portion of the ventricle at 6 dpf (∼55%). Thanks to the regenerative potential and cardiomyocyte fate plasticity of the zebrafish heart, the uninjured *tbx5a*-negative second heart field (SHF) cardiomyocytes compensate the ablation of the FHF-derived part of the ventricle (Sánchez-Iranzo et al., 2018), and regenerates the heart up to 85% of its full size in 3 days. This approach results in a robust and reproducible cardiac injury, as the same extent of ventricular cardiomyocyte injury is always induced by targeting only FHF-derived cells. The pool of remaining SHF-derived cardiomyocytes, responsible for the regenerative response, is also comparable in size among individuals. Combining this pharmaco-genetic approach with a state-of-the-art automated microfluidic bioimaging system makes the platform ideal for conducting pharmacological and genetic screens to identify modulators of cardiac regeneration. By leveraging this innovative approach, we aim to pave the way for the screening and discovery of drugs capable of enhancing heart regeneration in humans, ultimately offering alternative therapeutic approaches to heart failure.

## 2 Materials and equipment

### 2.1 Zebrafish lines, maintenance, and reagents

*Tg(tbx5a:CreERT2)* cn3Tg*; (myh7l:loxP-tagBFP-STOP-loxP-mCherry-NTR)* cn5Tg (Sánchez-Iranzo et al., 2018; https://zfin.org/ZDB-ALT-161004-1, https://zfin.org/ZDB-ALT-170711-2).

Wild type (WT) AB line, European Zebrafish Resource Center (EZRC).

*Tg(myl7:GFP)* line (Huang et al., 2003; https://zfin.org/ZDB-TGCONSTRCT-070117-164).

E3 zebrafish embryo medium (5 mM NaCl, 0.17 mM KCl, 0.33 mM CaCl_2_, 0.33 mM MgSO_4_; pH adjusted to 7.2).

Petri dishes 90 × 14 mm (Deltalab).

N-Phenylthiourea (Sigma-Aldrich, P7629).

### 2.2 Genetic ablation reagents and equipment

(Z)-4-Hydroxytamoxifen (tamoxifen; Sigma-Aldrich, H7904).

Metronidazole (Sigma-Aldrich, M3761).

Ethanol absolute, for analysis, ExpertQ^®^ (Scharlab, ET0005).

Dimethyl sulfoxide (DMSO; Sigma-Aldrich, D8418).

Molecular filtration chemical hood KIM Activa (Aquaria Srl).

ThermoMixer C (Eppendorf).

Leica M165 FC fluorescence stereo microscope.

### 2.3 Automated imaging system and reagents

VAST system (Union Biometrica) consisting of LP Sampler and VAST BioImager (Pardo-Martin et al., 2010).

Leica DM6B upright widefield microscope with a motorised Z-stage.

Leica DFC9000 GTC (4.2 megapixel) CMOS camera.

Leica Application Suite X software version 3.6 (Leica).

96 well plate ClearLine (DDBioLab).

Ethyl 3-aminobenzoate methanesulfonate salt (Tricaine; Sigma-Aldrich, A5040; pH adjusted to 7.2).

### 2.4 Video processing and analysis

Fiji software (Schindelin et al., 2012) with Bio-Formats plugin (Open Microscopy Environment consortium; Linkert et al., 2010).

ZeCardio^®^ software (Dyballa et al., 2019).

### 2.5 Drug screening system

Molecular filtration chemical hood KIM Activa (Aquaria Srl).

Dimethyl sulfoxide (DMSO; Sigma-Aldrich, D8418).

Alfacalcidol (Selleckchem, S1468).

Tyrphostin AG1478 (Merck, T4182-5 MG).

GSK1016790A (Merck, G0798).

### 2.6 CRISPR/Cas9 system and reagents and validation through sequencing

Ensembl Release 110 (July 2023; Martin et al., 2023).

Geneious Prime software, version 2023.2 (Biomatters; available from https://www.geneious.com).

CRISPOR (Concordet and Haeussler, 2018; available from http://crispor.tefor.net/).

Primer-BLAST (NCBI; Ye et al., 2012).

ThermoMixer C (Eppendorf).

Leica S Apo Stereozoom stereo microscope.

Microinjector PV850 (World Precision Instruments) with an external pressure source.

Microinjection needles (3 in, OD 1.0 mm, No Filament, World Precision Instruments, 1B100-3).

Microloader™ tips, 0,5–20 μL, 100 mm (Eppendorf, 5242956003).

Gene of interest-specific Alt-R^®^ CRISPR-Cas9 crRNAs, 2 nmol (IDT Technologies).

• Trpv4_ex1_sg2: TCT​ACT​GAA​GCG​CTA​TCG​TC
• Trpv4_ex1_sg4: TCC​GAC​TCA​AGA​AGA​TCC​AT

Alt-R^®^ CRISPR-Cas9 Negative Control crRNA #1, 2 nmol (IDT Technologies).

Alt-R^®^ CRISPR-Cas9 Negative Control crRNA #2, 2 nmol (IDT Technologies).

Alt-R^®^ CRISPR-Cas9 tracrRNA, 100 nmol (IDT Technologies, 1072534).

Alt-R^®^ *S.p.* Cas9 Nuclease V3, 500 ug (IDT Technologies, 1081059).

Nuclease-Free Duplex Buffer (IDT Technologies).

UltraPure™ DNase/RNase-Free Distilled Water.

Potassium chloride (Sigma-Aldrich, P9333).

CoboXtract™- Quick DNA Extraction Solution 1.0 (COBO scientific, C20101).

GoTaq^®^ G2 Flexi DNA Polymerase (Promega, M7801).

dNTPs NZYMix 10 mM (Nzytech, MB08605).

Gene of interest-specific PCR primer (IDT Technologies).

• Trpv4_ex1_Fw: CAC​CTG​GAC​ACA​TTC​CAG​AGT​AAT
• Trpv4_ex1_Rv: AGC​TTC​CTG​ACC​AAC​TTC​CTT

GreenSafe Premium (Nzytech, MB13201).

C1000 Touch Thermal Cycler with Dual 48/48 Fast Reaction Module (Bio-Rad).

QIAquick PCR Purification Kit (250) (Qiagen, 28106).

ICE CRISPR Analysis Tool (Synthego).

## 3 Methods

### 3.1 Animal husbandry and transgenic line

The double transgenic zebrafish line *tbx5a:CreERT2; myh7l:loxP-tagBFP-STOP-loxP-mCherry-NTR* (referred to as the Heartbreaker line hereinafter) was generated in WT AB background as described in Sánchez-Iranzo et al., 2018. Hemizygous larvae were used in the experiments. Larvae were maintained at a water temperature of 28°C in a 14 h light/10 h dark cycle, unless they were incubated with light-sensitive drugs, in which case they were kept in 24 h darkness.

The Heartbreaker line carries a transgenic cassette operating under two promoters: *myh7l* (*myosin heavy chain 7-like*, also known as *vmhcl*), which is specific to ventricular cardiomyocytes, and *tbx5a* (*T-box transcription factor 5a*), active in a more specific pool of FHF-derived ventricular cardiomyocytes [Supplementary Figure S1]. The genetic system utilises CreERT2, a Cre recombinase fused to mutant estrogen ligand-binding domain (ERT2) which requires the presence of tamoxifen for activation (Feil et al., 1997). This allows for tamoxifen-induced activation of Cre recombinase only in *tbx5a*-positive cells. The Cre activation leads to the excision of the genetic sequence between two loxP sites, leading to a subsequent induction of expression of nitroreductase (NTR). NTR is a bacterial enzyme that catalyses the reduction of the non-toxic prodrug Metronidazole (MTZ) into a cytotoxic product that triggers cell death (Curado et al., 2007). Therefore, the treatment of the Heartbreaker zebrafish larvae with tamoxifen and subsequently MTZ induces cell death specifically in NTR-expressing cells.

**FIGURE 1:**
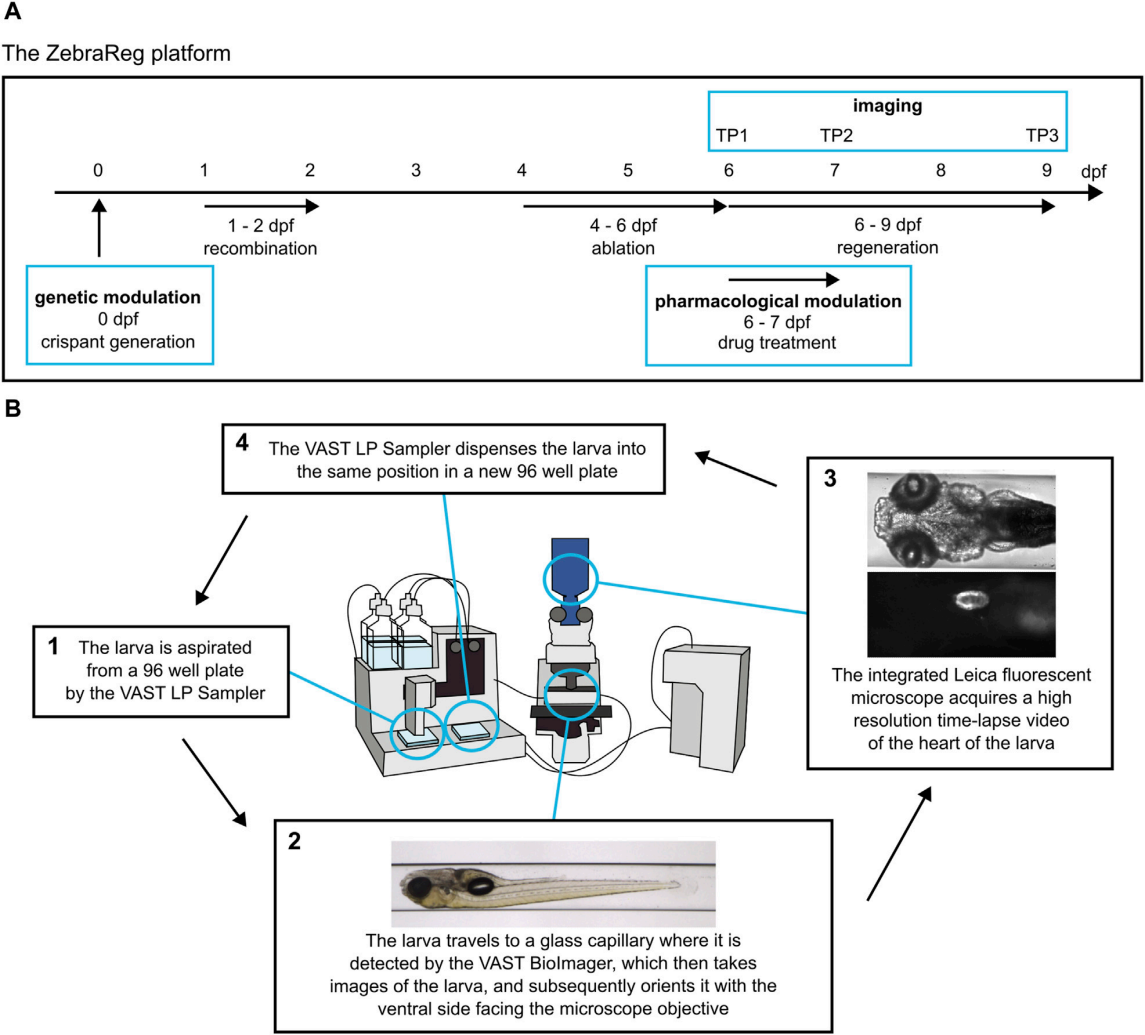
ZebraReg: a novel platform for discovering regulators of cardiac regeneration using zebrafish. **(A)** Overview of the timeline of the experimental procedure. The ZebraReg platform utilises the doubly transgenic Heartbreaker zebrafish line. In this zebrafish line, a tamoxifen-induced recombination and subsequent drug treatment lead to cell-specific death of a large pool of ventricular cardiomyocytes. Thus, a robust ventricular injury is caused, and is regenerated through the proliferation of the remaining cardiomyocytes. Automated imaging at six, seven and nine dpf (corresponding to timepoints TP1, TP2, and TP3) allows for the longitudinal analysis of the regenerative process. The process can be integrated with pharmacological and genetic approaches to determine the effect of genes and drugs on regeneration kinetics. In the case of pharmacological modulation, the drug treatment is performed for 24 h between TP1 and TP2. In the case of genetic modulation, a CRISPR/Cas9-based approach for generating F0 crispants is performed at one-cell stage to induce the loss of function of the gene of interest. **(B)** The automated imaging system consists of the VAST BioImager and LP Sampler and an integrated Leica fluorescent microscope for high resolution imaging. The systems cooperate in order to detect and image each larva. For each larva, two readouts are generated: firstly, a set of *in toto* images is obtained by the VAST BioImager for morphological readouts. The larva is then oriented with the ventral side facing the objective of the integrated fluorescent Leica microscope. Subsequently, the Leica microscope obtains a high-resolution time-lapse video of the heart. This video is then used to determine the size of the heart at each timepoint to determine the regeneration kinetics of each larva. The larva is then dispensed into a destination 96 well plate in the same position it was located in the source 96 well plate. Modified from Dyballa et al. (2019).

An important feature of the Heartbreaker line is the coupling of states of expression to fluorescent labels. The possible combinations of tamoxifen and MTZ treatments (none; tamoxifen only; tamoxifen and MTZ) stratifies the larvae into three distinct groups (negative control; not ablated; and ablated, respectively) based on the fluorescent label expression and presence of ventricular cardiomyocyte ablation. In the “negative control” group, all ventricular cardiomyocytes of this line express BFP and are therefore blue. In the case of the administration of tamoxifen (*i.e.*, “not ablated” group), Cre is activated in the *tbx5a*-positive cells, the FHF-derived portion of the ventricle, and undergo a colour switch from blue (BFP) to red (mCherry). SHF-derived *tbx5a*-negative cells remain blue. Thus, recombined larvae not treated with MTZ express both BFP and mCherry, in the SHF and FHF, respectively. Because the mCherry fluorophore is fused directly to the NTR, all and only mCherry-positive cells can catalyse MTZ and undergo apoptosis through genetic ablation. Thus, upon administration of MTZ, the “ablated” group shows specific ablation of the mCherry positive cells, while the BFP-positive *tbx5a*-negative cells, which are not sensitive to ablation, remain. The remaining untargeted BFP-positive portion of the ventricle will proliferate and reconstruct the heart during regenerative processes [Supplementary Figure S1]. An overview of the timeline of the experimental procedure is summarised in Figure 1A.

### 3.2 Induction of Cre recombination

Zebrafish embryos were obtained by mating individual homozygous adult Heartbreaker fish with WT AB fish through standard methods. The hemizygous progeny was collected at 0 hpf in E3 medium in Petri dishes. Unfertilised embryos were removed at 6 hpf.

5 mM tamoxifen stock solution was prepared by dilution in 100% EtOH and kept at −20°C protected from ambient light for a maximum of 2 months.

At 24 hpf, embryos were divided into two experimental groups: one to be treated with tamoxifen, and one untreated negative control group. The tamoxifen stock solution was heated up for 10 min at 65°C using a ThermoMixer to increase its activity (Felker et al., 2016). It was then diluted to 10 µM final concentration in E3 medium containing 0.2 mM N-Phenylthiourea (PTU) to reduce pigmentation (Karlsson et al., 2001). The treated group was treated with the tamoxifen solution, while the negative control group was treated with 0.2 mM PTU in E3 medium only. The plates were wrapped in aluminium foil to protect them from light and placed in the incubator overnight.

At 48 hpf, the tamoxifen was removed from the treated group. All embryos were washed 3 times in E3 medium and incubated in 0.2 mM PTU in E3 medium.

At 80 hpf, embryos treated with tamoxifen were screened for ventricular mCherry expression using the fluorescence stereo microscope.

### 3.3 Genetic ablation

1M stock solution of MTZ was prepared in 100% DMSO and kept at room temperature, protected from ambient light.

At 4 dpf, the tamoxifen-treated mCherry-positive larvae were divided into desired experimental groups, leaving a “not ablated” control group which would not be treated with MTZ. The negative control group and the not ablated control group were treated with 1% (v/v) DMSO and 0.2 mM PTU in E3 medium. The experimental plates for ablation were treated with 10 mM MTZ dissolved in 0.2 mM PTU in E3 medium to induce genetic ablation of NTR-expressing cardiomyocytes. The plates were wrapped in aluminium foil to protect them from light. At five dpf, the treatment was repeated for each group.

At six dpf, the MTZ solution was removed, the larvae were washed 3 times in E3 medium and incubated in 0.2 mM PTU in E3 medium. The larvae were then immediately prepared for the first timepoint of the automated imaging process.

### 3.4 Automated imaging of heart regeneration

The readout of regenerative kinetics is obtained using an automated microfluidic system consisting of VAST LP Sampler and BioImager (Pardo-Martin et al., 2010), and an integrated Leica fluorescent microscope, which has been proven to be suitable in high-throughput screening applications, such as cardiotoxicity analysis in zebrafish larvae (Dyballa et al., 2019). This integrated system allows for the processing of larvae from 96 well plates, their detection and automated orientating in a glass capillary, and high-resolution video and image acquisition with an external microscope [Figure 1B; modified from Dyballa et al., 2019]. After imaging, the larvae are returned to the same position in the 96 well plate, meaning that individual larvae can be tracked over time.

The first imaging timepoint (TP1) takes place at six dpf and corresponds to the timepoint immediately after injury, at 0 days post injury (dpi). Individual larvae were plated in 96 well plates and anaesthetised with 200 µL of 0.28 mg/mL solution of Ethyl 3-aminobenzoate methanesulfonate salt (tricaine) in E3 medium. The larvae were then imaged using the automated platform. For ventrally oriented imaging, the rotation angle for imaging with an external device was set to 180° and the lateral displacement was set to 600 µm in VAST BioImager software. The two systems cooperate in the imaging process; after sending a signal that the larva has been correctly recognised and positioned, the LASX software controlling the Leica microscope receives the signal and triggers automated image and video acquisition. The sequence of imaging events consists of the Autofocus function in the BFP channel, followed by video acquisition in the RFP and BFP channels, respectively. Timelapse videos were acquired with a 10× immersion water-dipping objective at binning 2 × 2 (effective pixel size 1.3 µm) at a frame rate of 96.8 frames per second (fps). Each video was captured for a duration of 2.8 s and saved in.lif format. After imaging, larvae were automatically dispensed into a new 96 well plate in the original position. They were then incubated with 200 µL of 0.2 mM PTU in E3 medium overnight.

Automated imaging process is repeated at seven dpf (TP2, one dpi) and nine dpf (TP3, three dpi) in order to capture regeneration dynamics. After the last imaging timepoint, larvae were sacrificed in 4 mg/mL tricaine stock solution in E3 medium.

### 3.5 Video analysis

Video analysis was performed in Fiji (Schindelin et al., 2012). Files in.lif format were imported into Fiji and time-lapse videos in mCherry and BFP channels opened as a virtual hyperstack using the Bio-Formats plugin (Linkert et al., 2010). When analysing regeneration kinetics, only the BFP channel videos were used, as it is the BFP-positive cells that repopulate the injured ventricle through regenerative processes.

During the 2.8 s long video acquisition, approximately 10 full heart beats are recorded. Therefore, readouts of both maximum and minimum ventricle size can be obtained. Minimum ventricle size during systole was chosen as the more reliable readout due to a stronger fluorescent signal. A video frame depicting complete heart systole was duplicated from the stack and converted to 8bit image. Gaussian blur filter (σ = 2) was applied to the image to reduce noise. Signal thresholding values were determined based on the fluorescence levels of negative control larvae at each experimental timepoint and used to analyse all videos in each individual experimental run. These values were set in such a way that the generated ROI encompassed the entire ventricle while excluding any nearby autofluorescent structures [Supplementary Figure S2A]. In case of presence of BFP signal in bulbus arteriosus, this area was discarded, and only ventricular BFP-positive areas were analysed. The physical size of the BFP positive area of the ventricle was obtained by measuring the size of the ROI obtained through thresholding in µm^2^. An.ijm macro can be generated with ease to partially automatise the process [Supplementary Material].

When analysing the mCherry-positive portions of the ventricle for validation purposes, an analogous approach was used, only using the mCherry series in not ablated control larvae to determine signal thresholding values (data not shown).

### 3.6 Data analysis

BFP positive areas of each experimental group were normalised at each timepoint to the negative control (mean = 100). The normalised BFP positive area values with the standard error of the mean (SEM) were plotted for each experimental timepoint to determine regeneration kinetics of each experimental group. The ROUT method (Q = 1%) in GraphPad Prism version 8.0.1 for Windows was used to remove outliers. For the setup experiments evaluating the ablation efficiency and regeneration kinetics, ordinary Two-way ANOVA followed by Tukey-Kramer’s multiple comparisons test was performed using GraphPad Prism version 8.0.1 for Windows to compare the three experimental conditions (negative control, not ablated, ablated) at each timepoint (TP1, TP2, TP3). Normality of residuals was tested through Anderson-Darling test and Shapiro-Wilk test. For the validation experiments evaluating the effects of drug treatment and gene knockout on regeneration kinetics, Anderson-Darling test was performed to test for normality and an unpaired *t*-test was performed using GraphPad Prism version 8.0.1 for Windows to compare the experimental condition to the ablated control.

### 3.7 Drug treatment

Alfacalcidol (Alfa) and GSK1016790A (GSK101) were used as pro-regenerative control compounds (Han et al., 2019; Yang et al., 2023), and AG1478 as an anti-regenerative control compound (Gemberling et al., 2015). Alfa was dissolved in DMSO to make a 10 mM stock solution and used at a final concentration of 5 µM as previously established in Han et al., 2019. GSK101 was dissolved in DMSO to make a 10 mM stock solution and used at a final concentration of 0.8 µM as determined as the BMD10 through a teratogenicity and acute developmental toxicity assay. AG1478 was dissolved in 100% molecular grade ethanol to make a 10 mM stock solution and used at a final concentration of 4.6 µM as determined as the BMD10 through a teratogenicity and acute developmental toxicity assay. Stock solutions of GSK101 and Alfa were kept at −80°C, AG1478 at −20°C.

All drugs were administered through direct dissolution in 0.2 mM PTU in E3 medium in a total DMSO concentration of 0.3% (v/v) on the day of the experiment. After automated imaging at TP1, larvae were treated with 200 µL of corresponding drug solution or 0.3% DMSO in 0.2 mM PTU in E3 control solution and incubated for 24 h while protected from ambient light. Drug solution was then removed and replaced with 200 µL of 0.28 mg/mL tricaine in E3 medium for anaesthesia. TP2 automated imaging was then performed as described previously.

### 3.8 Generation and confirmation of F0 crispants

The Alt-R CRISPR-Cas9 System by IDT was used to generate F0 crispants with a large deletion in the first exon of the *trpv4* gene. Two crRNAs targeting distinct sites of the exon were designed using CRISPOR (Concordet and Haeussler, 2018). Commercially available negative control crRNAs (IDT) were used as scramble controls. Each crRNA was assembled to tracrRNA in Nuclease-Free Duplex Buffer (IDT) to a final concentration of 30 µM by heating up to 95°C for 5 min and allowing it to cool at room temperature for 5 min. CRISPR/Cas9 ribonucleoprotein (RNP) complex was assembled by combining Alt-R *S.p.* Cas9 Nuclease V3 (3.7 µM final concentration) and the two sgRNAs targeting *trpv4* or two scramble control sgRNAs (9 µM final concentration total) with KCl (0.2 M final concentration) acting as a buffer to avoid needle obstruction. The mixture was incubated at 37°C for 10 min and afterwards kept on ice for immediate microinjection.

F0 crispants were generated through the microinjection of the Cas9 RNP complex into the cytoplasm of the embryo at the one-cell stage. For the setup and confirmation experiments, WT AB line was used, and approximately 100 eggs were injected. Unfertilised eggs were removed at 6 hpf. At 24 hpf, bulk genomic DNA was extracted using 50 µL of CoboXtract from a pool of 25 embryos to determine CRISPR/Cas9 efficacy. The mixture was incubated at 70°C for 10 min and at 98°C for 10 min, after which the extracted DNA was diluted in 70 µL of sterile nuclease-free water. PCR using GoTaq polymerase with *trpv4* primers flanking the sites targeted by sgRNAs was performed and the samples were purified using the QIAquick PCR Purification Kit and eluted in 11 µL of sterile nuclease-free water. The reverse primer (Trpv4_ex1_Rv) was used for sequencing. Samples were sequenced using the STAB VIDA Sanger sequencing service. ICE CRISPR Analysis Tool (Synthego) was used to determine the Indel % and the KO-score as readouts of CRISPR-Cas9 efficacy.

### 3.9 ZeCardio^®^ analysis of F0 crispants

In order to assess heart morphology and function, F0 crispants were generated in the *Tg(myl7:GFP)* line (Huang et al., 2003) using the protocol described above. The *Tg(myl7:GFP)* line expresses GFP in the heart under the myocardium-specific *myl7 (myosin light chain 7)* promoter. This allows for the assessment of cardiovascular function *in vivo*. Approximately 120 homozygous *Tg(myl7:GFP)* eggs obtained from a massive incross were injected for both *trpv4* crispants and scramble control conditions, respectively; a control group of approximately 40 embryos was left uninjected. Unfertilised eggs were removed at six hpf. At 24 hpf, the embryos were treated with 0.2 mM PTU in E3 medium and maintained in 0.2 mM PTU in E3 medium until five dpf.

At five dpf, the larvae were plated in a 96 well plate and anaesthetised with 200 µL of 0.28 mg/mL solution of tricaine in E3. Automated imaging was then performed with rotation angle for imaging with an external device of 120° and lateral displacement of 600 µm. The sequence of imaging events consisted of the Autofocus function in the GFP channel, followed by video acquisition in the GFP channel. Timelapse videos were acquired with a 10× immersion water-dipping objective at binning 2 × 2 (effective pixel size 1.3 µm) at a frame rate of 96.8 fps for a duration of 20 s. Time-lapse videos were exported in.lif format.

The videos were converted from.lif format to.zecardio format and imported in the ZeCardio software for semi-automatic analysis. The software relies on the user to define the position of the atrium and the ventricle to build a kymograph, after which it automatically determines the following readouts: the heartbeat frequency for each chamber; ejection fraction; lengths of individual beats; linearly corrected QT interval (QT phase); and maximal and minimal diameter for each chamber (Dyballa et al., 2019). ZeCardio software streamlines the extraction of heart parameters from videos, radically reducing the time needed for processing and analyses; however, the software is not commercially available. A similar analysis could be performed by using kymographs and image measurement tools in Fiji software (Schindelin et al., 2012), or a suitable software among the open-source analysis tools (Ling et al., 2022).

The individual values with SEM were plotted using GraphPad Prism version 8.0.1 for Windows and ROUT method (Q = 1%) was used to remove outliers. After outlier removal, ordinary one-way ANOVA followed by Tukey-Kramer’s multiple comparisons test was performed to compare the three experimental conditions (not injected control, scramble control, *trpv4* crispants). Normality of residuals was tested through Anderson-Darling test and Shapiro-Wilk test.

## 4 Results

### 4.1 Cre recombination, genetic ablation, and regeneration readout

**FIGURE 2:**
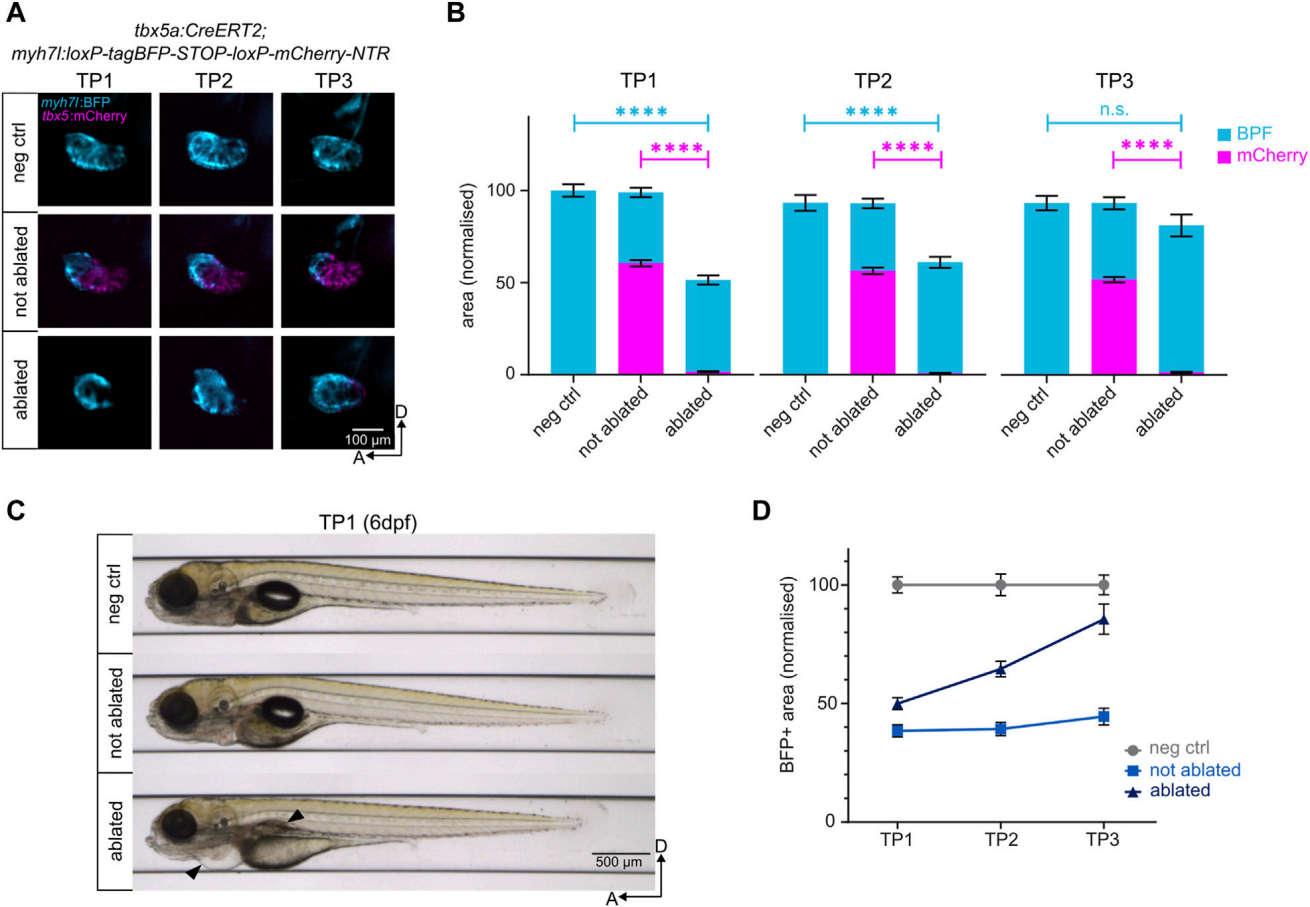
Genetic ablation of ventricular cardiomyocytes and subsequent regeneration. **(A)** Lateral view of the negative control, not ablated, and ablated ventricles. NTR-mCherry expressing cells undergo apoptosis in the ablated condition. The remaining BFP+ ventricular cardiomyocytes reconstruct the heart to levels comparable to the negative control by TP3. **(B)** Quantification of BFP+ and mCherry + areas of the ventricles after normalisation to negative control at each time point. At TP1, over 95% of NTR-mCherry expressing cells are ablated in the ablated condition. The BFP+ pool in ablated ventricles represents 45% of the BFP+ pool of the negative control ventricles. By TP3, it reaches approximately 85% of the BFP+ pool of the negative control ventricles. TP1: N = 35, 44, 42; TP2: N = 18, 39, 35; TP3: 14, 21, 19 for negative control, not ablated, and ablated larvae, respectively. **(C)** Automated imaging of negative control, not ablated, and ablated larvae at 6 dpf (TP1) through the VAST BioImager. Genetic cardiomyocyte ablation leads to the development of pericardial edema and swim bladder abnormality in ablated larvae (black arrows), consistent with a heart failure phenotype. **(D)** Regeneration kinetics graph. Representation of the BFP+ areas of the ventricles after normalisation to negative control at each time point.

Upon administration of tamoxifen to the hemizygous Heartbreaker embryos, approximately 25% induced Cre recombination (data not shown), displaying a colour switch from blue (BFP) to red (mCherry) in the FHF-derived ventricular cardiomyocytes. The proximal SHF-derived ventricle retained BFP expression [Figure 2A]. Recombined Heartbreaker larvae were then treated with MTZ to induce genetic ablation and cardiac damage. MTZ-driven genetic ablation led to a significant cardiomyocyte death, characterised by a loss of 97% of the mCherry-positive ventricle at TP1 [Figures 2A,B; Supplementary Figure S2B], and development of pericardial edema and swim bladder abnormality [Figure 2C], consistent with phenotypes associated with heart failure (Yue et al., 2015; Zhu et al., 2018). Approximately 50% of the total ventricle, corresponding to the SHF-derived cardiomyocyte pool, remains intact [Figures 2A,B; Supplementary Figures S2A,B]. This result confirms that the presented method induces a robust and reproducible ventricular cardiomyocyte-specific injury, while keeping a constant ventricular region uninjured.

Thanks to the automated imaging system provided by VAST, individual larval samples were imaged at the same anteroposterior position and dorsoventral orientation throughout three timepoints, thus allowing the evaluation of the regeneration kinetics of individual larvae [Supplementary Figure S2C,C’]. The longitudinal analysis of heart size revealed that the uninjured ventricular cardiomyocytes proliferate in response to the ventricular injury, leading to a gradual increase in ventricle size from 0 dpi (TP1) through one dpi (TP2) to three dpi (TP3). At TP2, the size of the ventricle increases from 50% to approximately 65% [Figure 2D]. By TP3, approximately 85% of the size of the negative control ventricle is restored in the ablated group, and the morphology of the regenerated ventricle resembles that of the negative control [Figures 2A,B,D]. Thus, the robust injury induced through genetic ablation is regenerated through the proliferation of remaining SHF-derived ventricular cardiomyocytes by three dpi (Sánchez-Iranzo et al., 2018).

### 4.2 Validation of the ZebraReg platform with pharmacological approaches

**FIGURE 3:**
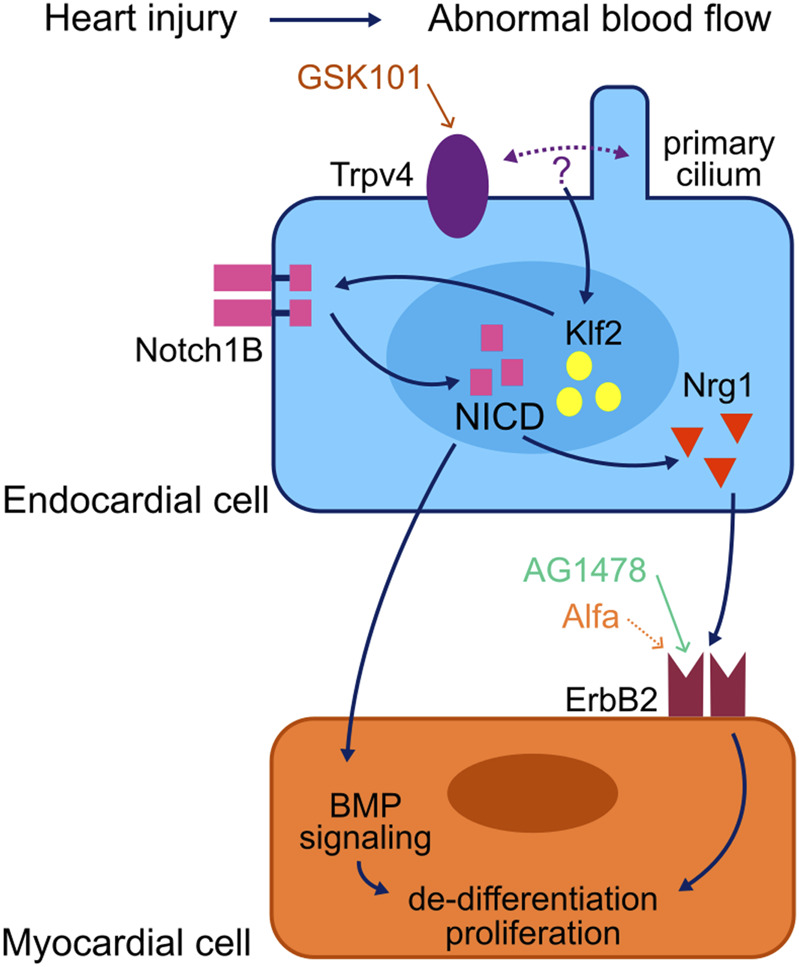
Model of a myocardial regeneration signalling network. Activation of the mechanosensory channel Trpv4 in the endocardium triggers a series of signalling events which lead to the activation of the ErbB2 channel in the myocardium and thus the initiation of regenerative processes in the injured myocardium. Adapted from Li et al. (2021).

In the current model of events triggering the injury-responsive regenerative processes in myocardial cells, non-cell autonomous signalling from the adjacent endocardium plays a critical role [Figure 3; adapted from Li et al., 2021]. In this model, the primary cilium and mechanosensory channels such as Trpv4 are present in the membrane of endocardial cells and protrude into the lumen of the heart where they sense shear stress forces generated by blood flow (Heckel et al., 2015; Andrés-Delgado and Mercader, 2016). An abnormal, oscillatory blood flow is generated in the zebrafish heart after ventricular injury, leading to the modulation of activity of the hemodynamic responsive transcription factor *klf2* through the activation of Trpv4 (Gálvez-Santisteban et al., 2019). This leads to the activation of endocardial Notch signalling, which is essential for the regeneration of the injured myocardium (Zhang et al., 2013; Gálvez-Santisteban et al., 2019; Zhao et al., 2019; Li et al., 2020; reviewed in Li et al., 2021).

Notch signalling supports cardiomyocyte proliferation by non-cell autonomous modulation of several myocardial signalling pathways; most notably the initiation of myocardial ErbB2 and BMP signalling (Gálvez-Santisteban et al., 2019). Neuregulin1 (Nrg1) has been identified as a potent mitogen for the endogenous adult zebrafish heart regeneration program (Gemberling et al., 2015). Nrg1 acts through its tyrosine kinase receptors ErbB2 and ErbB4 to promote de-differentiation and proliferation of cardiomyocytes, and ErbB2 inhibition blocks the regenerative processes (D’Uva et al., 2015; Gemberling et al., 2015). Vitamin D receptor (VDR) signalling has also been found to modulate cardiac regeneration, and vitamin D acts as a myocardial mitogen through ErbB2 (Han et al., 2019).

**FIGURE 4:**
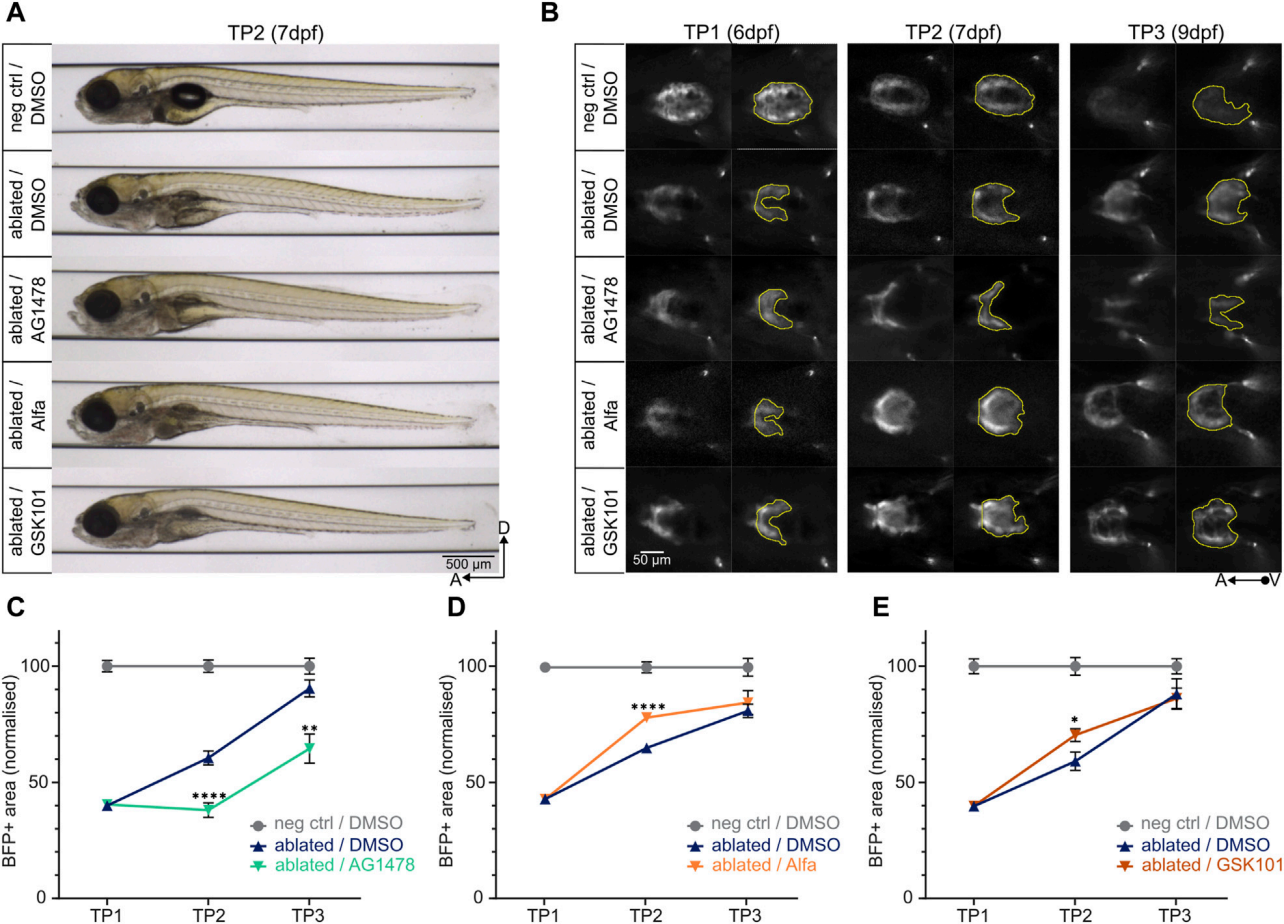
Determining the pro- and anti-regenerative effects of drugs on regeneration kinetics. **(A)** Larvae at TP2 after 24-h treatment with AG1478 (4.6 µM), Alfa (5 µM), or GSK101 (0.8 µM) do not show any significant developmental defects compared to the ablated control treated with 0.3% DMSO. **(B)** Representative images of ventral view of ventricles during systole (left) and BFP+ ventricular areas (right). At TP2, the treatment with AG1478 leads to a decrease in BFP+ ventricular area compared to ablated DMSO control; on the other hand, the treatment with Alfa and GSK101 leads to an increase. **(C)**, **(D)**, **(E)** Regeneration kinetics graphs after 24-h treatment with selected drugs between TP1 and TP2. Pro- and anti-regenerative effects of drugs can be assessed. For each condition, two separate experiments were performed, and values pooled after normalisation to corresponding negative control at each time point. **(C)** The treatment with AG1478 leads to a highly significant decrement in BFP+ area at both TP2 and TP3 compared to ablated DMSO control. TP1: N = 31, 31, 22; TP2: N = 24, 27, 22; TP3: N = 12, 16, 16 for negative DMSO control, ablated DMSO control, and AG1478 treated group, respectively. At TP2, *p* <0.0001; at TP3, *p* = 0.0012. **(D)** The treatment with Alfa leads to a highly significant increment in BFP + area at TP2 compared to ablated DMSO control. TP1: N = 32, 40, 29; TP2: N = 27, 29, 30; TP3: N = 12, 15, 16 for negative DMSO control, ablated DMSO control, and Alfa treated group, respectively. *p* <0.0001. **(E)** The treatment with GSK101 leads to a significant increment in BFP + area at TP2 compared to ablated DMSO control. TP1: N = 21, 35, 27; TP2: N = 17, 22, 20; TP3: N = 8, 11, 10 for negative DMSO control, ablated DMSO control, and GSK101 treated group, respectively. *p* = 0.0101.

In order to assess the efficacy and versatility of the ZebraReg platform, we used compounds affecting heart regeneration kinetics. Drug treatments were performed in the 24-h window between TP1 and TP2. AG1478 was used as an anti-regenerative control compound. AG1478 is a ErbB tyrosine kinase receptor inhibitor commonly used as an ErbB2 inhibitor in zebrafish (Han et al., 2019) and its administration has been shown to impair cardiomyocyte proliferation during regeneration (Gemberling et al., 2015). Administration of 4.6 µM AG1478 for 24 h between TP1 and TP2 did not lead to any developmental toxicity in the larvae [Figure 4A]; however, at TP2, a significant reduction of the BFP-positive ventricular area was observed [Figure 4B], suggesting a defect in regeneration in the AG1478-treated ablated hearts. Accordingly, the regeneration kinetics of AG1478-treated larvae was statistically significantly diminished compared to DMSO-treated ablated control at both TP2 and TP3 [Figure 4C; Supplementary Figure S3A].

As pro-regenerative control compounds we used Alfacalcidol (Alfa) and GSK1016790A (GSK101) (Han et al., 2019; Yang et al., 2023). Alfa is a vitamin D analogue that acts through ErbB2 to promote proliferation in zebrafish cardiomyocytes, and its administration has been shown to increase cardiomyocyte proliferation in regenerating zebrafish hearts (Han et al., 2019). Administration of 5 µM Alfa for 24 h between TP1 and TP2 did not lead to any developmental toxicity in the larvae [Figure 4A]. At TP2, a significant increase in BFP-positive ventricular area of the ablated hearts was observed in Alfa-treated larvae, consistent with its role in promoting cardiomyocyte proliferation after injury, compared to DMSO-treated ablated control [Figures 4B,D; Supplementary Figure S3B]. A similar effect was observed after the administration of 0.8 µM GSK101 [Figures 4A,B,E; Supplementary Figure S3C], a potent agonist of the mechanosensory channel Trpv4 (Thorneloe et al., 2008) with a demonstrated cardioprotective effect when administered after acute myocardial infarction in rats (Yang et al., 2023).

Thus, the ZebraReg platform is suitable for the detection of variation in cardiac regeneration abilities upon administration of compounds reducing and enhancing heart regeneration kinetics.

### 4.3 Using the ZebraReg platform to determine regeneration kinetics of crispant larvae

To validate the possibility of integrating the ZebraReg platform with CRISPR/Cas9-based genetic manipulation approaches, we generated crispants targeting *trpv4*, a gene coding for one of the mechanosensory channels upstream the activation of endocardial Notch signalling, required for ErbB2 activation in cardiomyocytes (Gálvez-Santisteban et al., 2019; Figure 3). *trpv4* loss of function has been associated with a diminished cardiomyocyte proliferative response to injury (Gálvez-Santisteban et al., 2019). sgRNAs were designed to target sequences in exon one of the *trpv4* gene, approximately 250 bp apart, to cause a large deletion in the first exon of the gene and thus a presumed loss of function [Supplementary Figure S4A]. The efficacy of mutagenesis in F0 crispants was assessed in WT AB line and confirmed through sequencing to be 95% [Supplementary Figure S4B].

**FIGURE 5:**
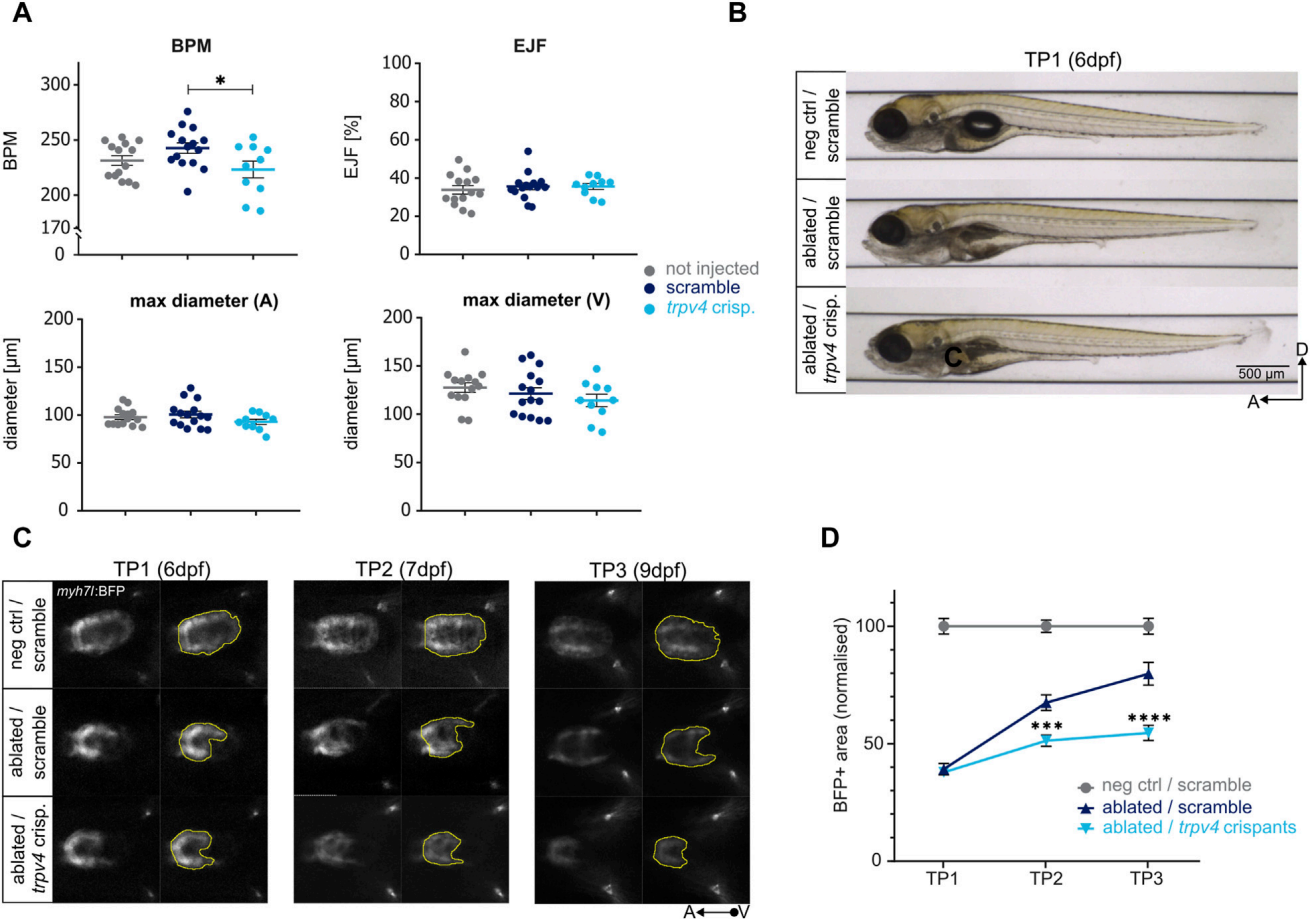
Determining the effect of genes on regeneration kinetics. The CRISPR/Cas9-driven loss of function of *trpv4* leads to an impaired regeneration. **(A)** The loss of function of *trpv4* does not lead to an overt cardiac development defect compared to non-injected and scramble-injected controls at 5dpf as seen in the following functional and morphological ZeCardio readouts: beats per minute (BPM), ejection fraction (EJF), and maximum diameter of the atrium (A) and ventricle (V). A significant reduction in BPM is observed compared to scramble control (*p* = 0.0456) but not non-injected control (*p* = 0.5635). N = 14, 15, 10 for not injected control, scramble control, and *trpv4* crispants, respectively. **(B)** The loss of function of *trpv4* does not lead to any overt developmental defects compared to scramble-injected ablated larvae at 6dpf (TP1). **(C)** Representative images of ventral view of ventricles during systole (left) and BFP + ventricular areas (right) at TP1, TP2, and TP3. The loss of function of *trpv4* leads to a decrease in BFP+ ventricular area compared to scramble-injected ablated control at both TP2 and TP3. **(D)** Regeneration kinetics graph. The loss of function of *trpv4* leads to a highly significant decrement in BFP+ area at both TP2 and TP3 compared to scramble-injected ablated control. Two separate experiments were performed, and values pooled after normalisation to corresponding negative control at each time point. TP1: N = 24, 27, 35; TP2: N = 16, 20, 31; TP3: N = 8, 18, 27 for negative scramble-injected control, ablated scramble-injected control, and ablated trpv4 crispants, respectively. At TP2, *p* = 0.0002; at TP3, *p* <0.0001.

Prior to inducing genetic ablation, we used the ZeCardio software, a semi-automated software which determines morphological and functional readouts from time-lapse heart videos, to test possible cardiac development defects induced by the loss of *trpv4*. No changes were observed at the morphological level (*i.e.*, maximum diameter of atrium and ventricle), nor in the ejection fraction of *trpv4* crispants. A slight but significant reduction in the heart rate was detected in *trpv4* crispants compared to scramble control, but not to non-injected control [Figure 5A; Supplementary Figure S4D], suggesting that overall, no relevant cardiac development defects are induced under physiological conditions by the loss of *trpv4.*

*trpv4* crispants did not exhibit any developmental defects at six dpf upon genetic ablation compared to scramble-injected ablated controls [Figure 5B]. At TP1, the BFP-positive ventricular areas of ablated *trpv4* crispants were identical in size to scramble-injected ablated controls. However, in contrast to scramble-injected ablated controls, which regenerated to up to 80% of the size of the scramble-injected negative control hearts by TP3, *trpv4* crispant hearts failed to regenerate [Figures 5C,D]. A highly significant decrement in regeneration kinetics was observed at both TP2 and TP3, pointing to a pronounced defect in the induction of pro-regenerative response in *trpv4* crispants [Figure 5D].

The ZebraReg platform can thus be utilised to assess the regeneration kinetics of both cardiac drugs and genetic mutants.

## 5 Discussion

Cardiovascular disease such as myocardial infarction is the leading cause of morbidity and mortality world-wide (World Health Organization, 2014). Currently, no therapy is available to revert the detrimental effects of myocardial infarction on the heart muscle. Many strategies have focused on cell-based approaches but have yet not been successful (van der Pol and Bouten, 2021). Therefore, an alternative approach aiming to enhance proliferation of residing cardiomyocytes has become of great interest (Laflamme and Murry, 2011; Garbern and Lee, 2022). This could be achieved through the targeting of modulators of cardiac regeneration by small molecules. Therefore, it is crucial to understand the molecular processes underlying cardiomyocyte regeneration to identify new druggable targets and test the effect of such compounds on heart regeneration (Sadek and Olson, 2020).

In the last decades, there has been a decline in productivity in biopharmaceutical industry, marked by a relatively low number of novel drugs entering the market due to a low clinical approval rate (Pammolli et al., 2011; Cornet et al., 2018; Cassar et al., 2020). Using zebrafish larvae in biopharmaceutical research can be beneficial in streamlining the drug discovery process by quickly eliminating candidate drugs with undesirable toxicity profiles (Cassar et al., 2020). Additionally, zebrafish larvae are compatible with the 3Rs (Replacement, Reduction, and Refinement) of animal models (Cornet et al., 2018; Cassar et al., 2020). The 3R are a framework of practices aiming to improve laboratory animal welfare through the Replacement of the use of laboratory animals with alternative tools, Reduction of the number of animals used for experimentation, and Refining of experimental methods to minimise pain and suffering. Therefore, we believe the ZebraReg platform can be a very beneficial tool for identifying novel druggable targets, as well as performing medium-throughput screens to discover compounds with pro-regenerative effects.

### 5.1 Strengths and limitations

Multiple advantages characterise the larval cardiomyocyte ablation model introduced here, ZebraReg, which includes a pharmacological and genetic validation. Firstly, the larval heart regenerates in a very precise and robust manner, allowing hearts that have lost ∼55% of their mass to reach a nearly full recovery in size and morphology in a matter of 3 days. Secondly, according to our pharmacological and genetic validation, the larval heart activates similar regenerative pathways as those described in adult heart regeneration (*i.e.*, ErbB2 signalling), being a suitable model for the testing of drugs with potential applications in adult patients. Finally, the evaluation of kinetics of regeneration and significant differences between ablated and non-ablated samples allow evaluating both pro- and anti-regenerative molecules and targets. To our knowledge, this is the first drug screening platform detecting specific effects on cardiac regeneration after injury, as opposed to evaluating cardiac regeneration based on studying endogenous cardiomyocyte proliferation (Choi et al., 2013).

Although the approach proposed is based on induction of cardiomyocyte ablation at 96 hpf, a stage where a two chambered heart is already formed and functional (Matrone et al., 2013; Brown et al., 2016), cardiac regeneration during the analysed time frame might be due to a mixed contribution of genetic developmental programs and injury-driven gene activation. As expected, transcriptomic profiling has shown that a high number of genes upregulated in the zebrafish larval heart – compared to the adult zebrafish heart most commonly used for regeneration studies – are involved in cell proliferation (Shih et al., 2015). Interestingly, it has been recently shown that adult cardiomyocytes are genetically heterogeneous, and some populations express embryonic markers of immature cardiomyocytes upon injury (Tsedeke et al., 2021). The highly dynamic transcriptional landscape and highly proliferative state of larval cardiomyocytes is likely to boost and accelerate cardiac tissue recovery in embryonic injury models. Nonetheless, there are some features which are specific to the regenerative process such as an initial inflammatory response, removal of necrotic tissue, and neovascularization, which are less relevant during embryonic development but are crucial for providing nutrients and removing waste during tissue repair.

ZebraReg can be defined as a medium- and not high-throughput screening method because of the time limiting factor of the imaging step. The imaging process of a 96-well plate takes approximately 3.5 h. In a plate it is possible to test up to five compounds per day, with 24 larvae per condition, including all control groups. This translates into being able to run two 96-well plates in one day. Thus, when compared with other zebrafish larval screenings, which might allow testing dozens of compounds per day, ZebraReg stands as a medium-throughput screening method.

In this study, we validated the pro-regenerative effect of the Trpv4 mechanosensory channel agonist GSK101 in zebrafish larvae. GSK101 has been found to promote angiogenesis and have a cardioprotective effect in rat acute myocardial infarction model (Yang et al., 2023). However, its pro-regenerative mode of action in our model might be different; in contrast to the rat model, in our genetic ablation approach, it is only cardiomyocytes that are injured and all other cardiac components, including vasculature, remain intact (Wang et al., 2011). In the zebrafish, *trpv4* null mutants exhibit reduced cardiomyocyte proliferation and overall impaired recovery after ventricular injury (Gálvez-Santisteban et al., 2019). After cardiac injury, an abnormal blood flow leads to the activation of endocardial Trpv4 and a downstream non-cell autonomous activation of pro-regenerative processes in the injured myocardium (Zhang et al., 2013; Gálvez-Santisteban et al., 2019; Zhao et al., 2019; Li et al., 2020; reviewed in Li et al., 2021). We hypothesize that in our model, GSK101 exerts its positive effect on heart regeneration through the direct stimulation of endocardial Trpv4 channels, leading to an increased cardiomyocyte proliferative in response to injury. Trpv4 is recognised as a druggable target in pulmonary edema and congestive heart failure; however, so far, only GSK2798745, a Trpv4 antagonist, has entered clinical trials (Thorneloe et al., 2012; Lawhorn et al., 2021). Our results suggest that modulating *trpv4* activity in a cardiac injury setting may be a therapeutic approach worthy of further investigation.

We propose the ZebraReg platform as a possible tool for the discovery of new druggable targets through the CRISPR/Cas9-driven targeted disruption of genes of interest. In recent years, the innovations in CRISPR/Cas9 approaches in the zebrafish have allowed for up to a 100% mutagenesis rate in injected embryos (Varshney et al., 2015). This has made the use of crispants, or F0 mutants, possible. The advantage of using crispants, as compared to stable isogenic mutant lines, lies in the fact that the same larvae that were injected with the CRISPR/Cas9 system are directly analysed without the need to wait for the growth of multiple generations (Rubbini et al., 2020). However, a relatively large number of eggs (∼160) need to be injected with the CRISPR/Cas9 system targeting the gene of interest, as only approximately 25% of Heartbreaker hemizygous progeny induced Cre recombination upon tamoxifen treatment (data not shown).

All these biological and experimental features make ZebraReg suitable for discovering new therapeutic targets and drugs. Importantly, it might allow complementing zebrafish adult infarct models, which display a much lower experimental throughput and require extensive technical experience for their exploitation (González-Rosa, 2022). As such, the ZebraReg platform can be used for medium-throughput screens of targets and compounds with an unknown effect on regeneration.

### 5.2 Conclusion

In this manuscript, we present ZebraReg, a novel platform for studying heart regeneration using zebrafish larvae. A pharmaco-genetic approach is utilised to induce cell death in a specific sub-population of ventricular cardiomyocytes on a massive scale. This in combination with a medium-throughput automated imaging system allows for the longitudinal analysis of regeneration kinetics. It is a versatile method that can be integrated with pharmacological and genetic approaches and can be used to identify both pro- and anti-regenerative effects of genes and drugs. Using this platform, we hope to pave the way for discovering new druggable targets and drugs to offer alternative therapeutic approaches to myocardial infarction.

## Data Availability

The raw data supporting the conclusion of this article will be made available by the authors, without undue reservation.
