# Supplementary material for: ZebraReg—a novel platform for discovering regulators of cardiac regeneration using zebrafish
Source: Front Cell Dev Biol. 2024 May 10;12:1384423. doi: 10.3389/fcell.2024.1384423 (PMC11116629; doi:10.3389/fcell.2024.1384423)
Supplement: Supplementary file 1 [file DataSheet1.PDF]

Supplementary Material

1 Supplementary Figures

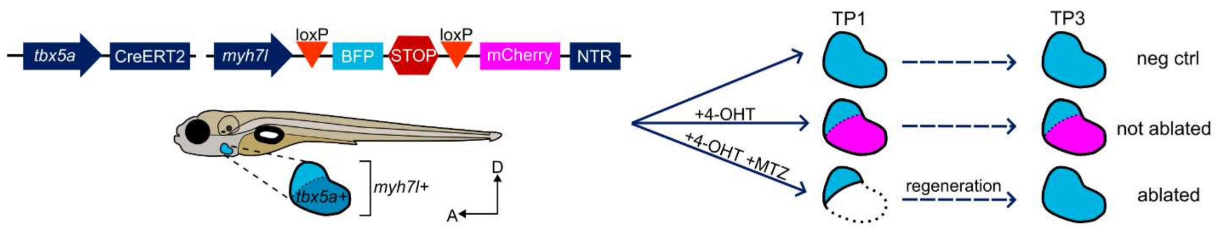

Supplementary figure S1. ZebraReg: a novel platform for discovering regulators of cardiac regeneration using zebrafish. The pharmaco-genetic ablation approach relies on a double transgenic system. The Heartbreaker zebrafish line carries two transgenes which cooperate in inducing a robust cardiomyocyte injury. The specific ablation of cardiomyocytes is achieved through the tamoxifen-induced expression of nitroreductase (NTR) fused to mCherry in *tbx5a+* subpopulation of *myh7l+* ventricular cardiomyocytes. Subsequent treatment with the antibiotic metronidazole (MTZ) induces cell death specifically in *tbx5a+* NTR-mCherry expressing cells at 0 days post injury (dpi). Individual larvae are followed from 0 to 3 dpi over three imaging timepoints (TP1, TP2, TP3) to assess regeneration kinetics.

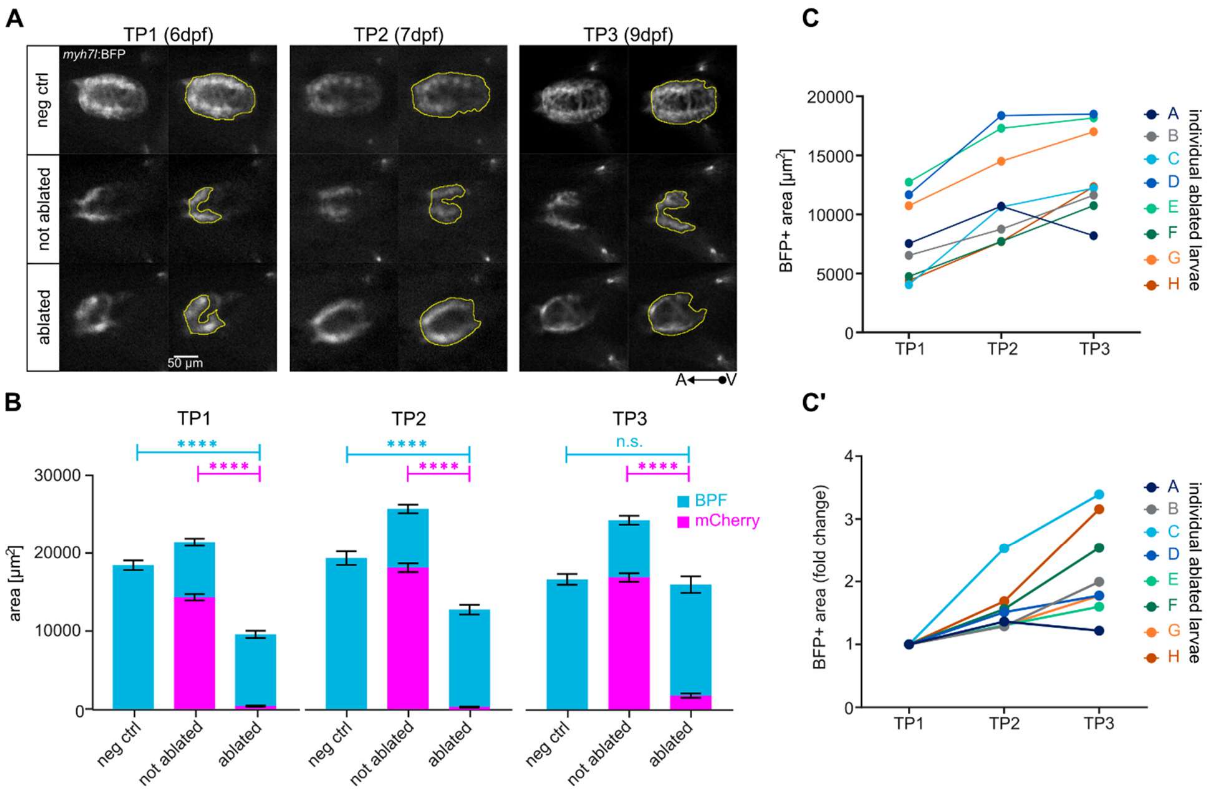

Supplementary figure S2. Genetic ablation of ventricular cardiomyocytes and subsequent regeneration. **(A)** Time-lapse acquisition through integrated fluorescent microscope. The larvae are positioned in the VAST BioImager such that the videos are acquired from the ventral side of the larvae. Representative images of ventral view of negative control, not ablated, and ablated ventricles during systole (left) and generation of ROI to measure BFP+ ventricular areas (right) are depicted. **(B)** Raw quantification of BFP+ and mCherry+ areas of the negative control, not ablated, and ablated ventricles in μm^2, obtained from automated time-lapse fluorescent imaging. TP1: N = 35, 44, 42; TP2: N = 18, 39, 35; TP3: 14, 21, 19 for negative control, not ablated, and ablated larvae, respectively. **(C)**, **(C')** Regeneration kinetics of individual ablated larvae (A – H). While the increment in BFP+ areas differs between individual larvae, all increase in size between TP1 and TP3. **(C)** Raw quantification of BFP+ ventricular areas of individual ablated larvae in μm^2. **(C')** BFP+ areas after normalisation to negative control at each time point and represented as fold change in size of each individual as compared to corresponding size at TP1.

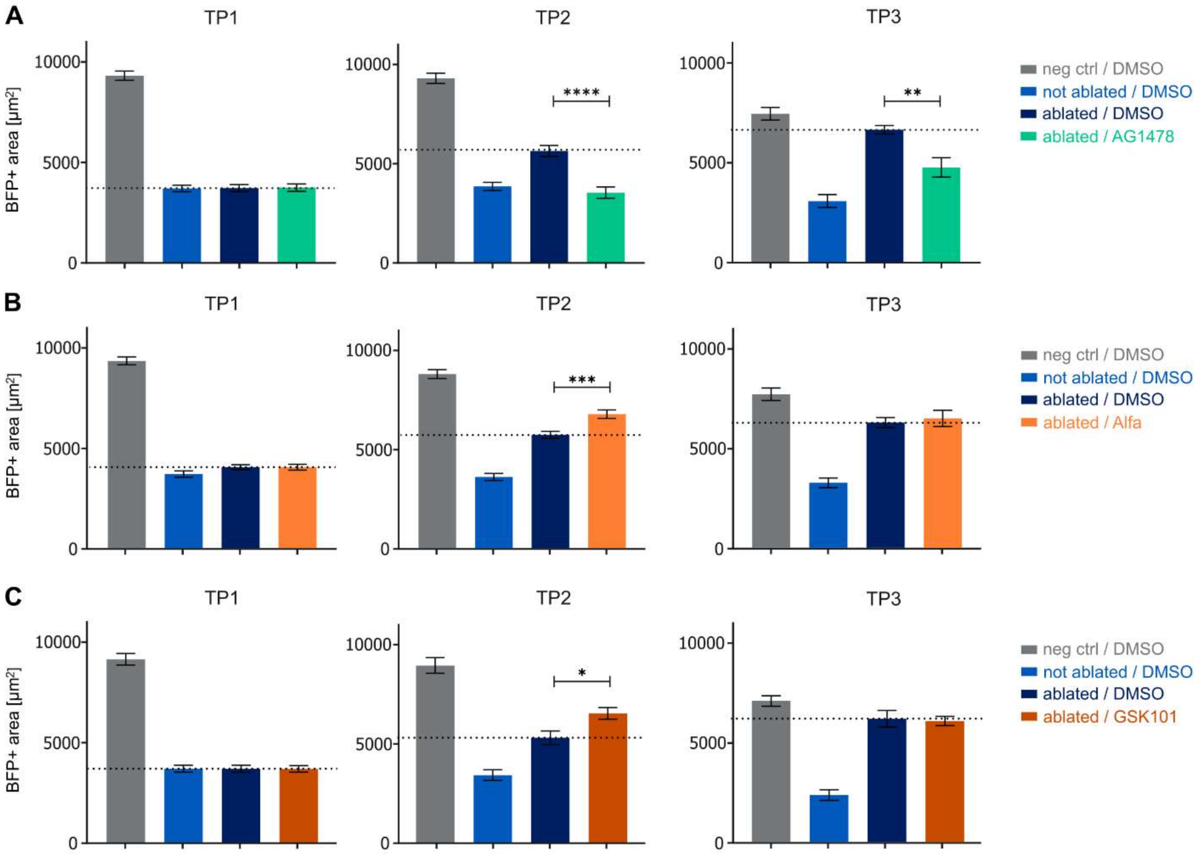

Supplementary figure S3. Determining the pro- and anti-regenerative effects of drugs on regeneration kinetics. **(A)**, **(B)**, **(C)** Raw quantification of BFP+ ventricular areas in μm^2. Two separate experiments were conducted for each drug treatment, and raw obtained values pooled. **(A)** The treatment with AG1478 leads to a highly significant decrement in BFP+ area at both TP2 and TP3 compared to ablated DMSO control. TP1: N = 31, 27, 31, 22; TP2: N = 24, 26, 27, 22; TP3: N = 12, 18, 16, 16 for negative DMSO control, not ablated DMSO control, ablated DMSO control, and AG1478 treated group, respectively. At TP2, $P < 0.0001$; at TP3, $P = 0.0012$. **(B)** The treatment with Alfa leads to a highly significant increment in BFP+ area at TP2 compared to ablated DMSO control. TP1: N = 32, 32, 40, 29; TP2: N = 27, 28, 29, 30; TP3: N = 12, 19, 15, 16 for negative DMSO control, not ablated DMSO control, ablated DMSO control, and Alfa treated group, respectively. $P < 0.0001$. **(C)** The treatment with GSK101 leads to a significant increment in BFP+ area at TP2 compared to ablated DMSO control. TP1: N = 21, 23, 35, 27; TP2: N = 17, 22, 22, 20; TP3: N = 8, 15, 11, 10 for negative DMSO control, not ablated DMSO control, ablated DMSO control, and GSK101 treated group, respectively. $P = 0.0101$.

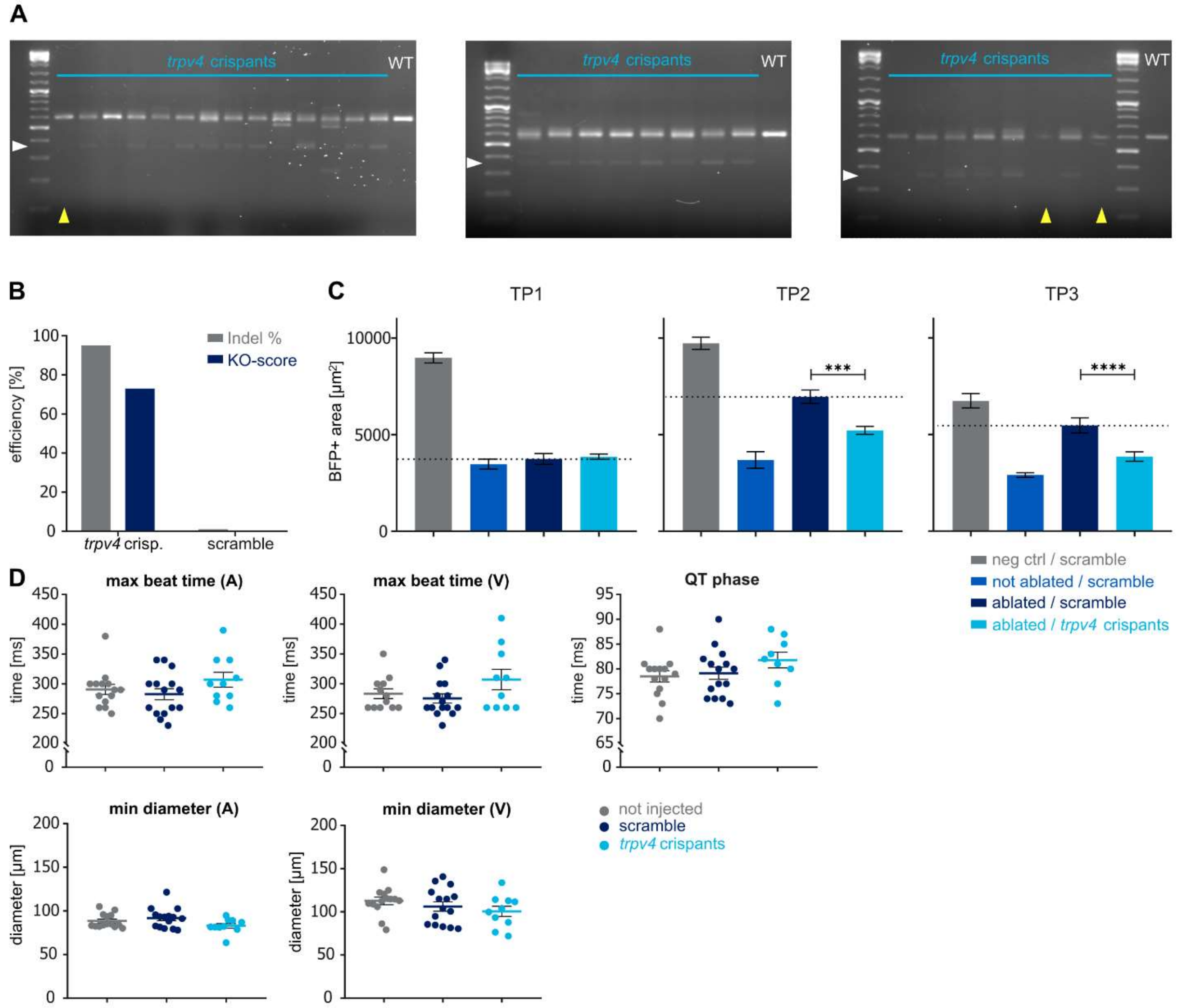

Supplementary figure S4. Determining the effect of genes on regeneration kinetics. **(A)** PCR amplifying the targeted region of exon 1 of *trpv4* on individual *trpv4* crispants used in the regeneration experiment versus wild type control. Two sequences 258 bp apart in exon 1 are targeted by the CRISPR/Cas9 machinery, leading to an excision of this portion of exon 1 in some cells, causing a loss of function of the gene (white arrow). Crispant larvae without the presence of the deletion band were excluded from further analysis (yellow arrow). **(B)** ICE analysis (Synthego) of Sanger sequencing of pools of 25 crispant larvae (1 dpf) injected with CRISPR/Cas9 system targeting *trpv4* or scramble control. The analysis shows that 95% of cells in *trpv4* crispants carry an indel mutation in the targeted loci in exon 1 of *trpv4*. **(C)** Raw quantification of BFP+ ventricular areas in µm^2. Two separate experiments were conducted, and raw obtained values pooled. TP1: N = 24, 26, 27, 35; TP2: N = 16, 13, 20, 31; TP3: N = 8, 9, 18, 27 for negative scramble-injected control, not ablated scramble-injected control, ablated scramble-injected control, and ablated *trpv4* crispants, respectively. **(D)** Additional functional and morphological ZeCardio readouts: maximum complete beat time of the atrium (A) and ventricle (V), QT phase (linear correction, k = 0.93), and minimum diameter of the atrium and ventricle. No significant difference in any readout is observed in *trpv4* crispants compared to non-

injected and scramble-injected controls. N = 14, 15, 10 for not injected control, scramble control, and *trpv4* crispants, respectively.

2 Supplementary material

```
setOption("ScaleConversions", true);
run("8-bit");
run("Gaussian Blur...", "sigma=2");
run("Threshold...");
setThreshold(55, 255, "raw");
doWand(255, 290);
run("Measure");
String.copyResults();
```

Supplementary code. An example of an .ijm macro for use in Fiji software to automatically measure the BFP+ ventricular areas. After conversion to an 8bit image, Gaussian blur is applied to the image to reduce noise. The same thresholding is applied to each image to generate a ROI which includes the entire BFP+ portion of the ventricle while excluding any nearby autofluorescent structures. The physical size of the BFP+ area of the ventricle was obtained by measuring the size of the ROI in μm^2.
